# Prevalence of HIV disease between Qeshm Island people during 2013-2014, Iran

**Published:** 2015

**Authors:** N Holakouie, A Kargar Kheirabad, MJ Sajjadi, H Gouklani

**Affiliations:** * Student Research Committee, Hormozgan University of Medical Sciences, Bandar Abbas, Iran,; ** Department of Virology, School of Public Health, Tehran University of Medical Sciences, Tehran, Iran,; *** Molecular Medicine Research Center, Hormozgan University of Medical Sciences, Bandar Abbas, Iran

**Keywords:** epidemiology, HIV infections, Qeshm

## Abstract

**Introduction:** Got safe loss syndrome (HIV) is represented with a variety of disorders of cellular and humoral immune dysfunction caused with personal immunodeficiency disease (HIV) infection. Immune deficiency caused by HIV, leads to opportunistic diseases & certainly the progression of the infections cause the patient’s death. That is why we chose to realize this research, to assess the prevalence of HIV among the Qeshm Island inhabitants.

**Materials and Method:** The cross-sectional research did carry on 1500 subjects. The sampling approach is the stratify-cluster compound. Ten head-clusters were randomly selected from each center and individuals are received from within the blocks. Later developing the questionnaire, including demographic the information and danger agents, gore examples are captured from the brachial vein. The currency of HIV-Ab is assessed with the approach of ELISA. Indeed, the actuarial studies are performed with applying the actuarial Plan for public Sciences software (SPSS) program issue 16.0. The information is examined with Chi-square and detailed actuarial trial.

**Results:** The all over the currency of HIV disease is zero. Of the members in the examination, 511 (34.1%) are men and 989 (65.9%) were women. This age of mediocre is 32.6 years. 88% and 12% of the individuals were married and single, respectively. The training plane of maximum cases (66%) was the degree diploma. In terms of location, mostly subjects (75.2%) lived into the village region. 136 (7.9%) had a history of travelling abroad and none of the subjects did not report a history of running away from home.

**Conclusion:** The most of the subjects lived in the rural area and were married women with high school education. Although there were cases that had records of sexually spread illnesses (STD) or tattoos, HIV prevalence was zero. This gives hope to the health of our society Regarding HIV disease.

## Introduction

AIDS reaches for received safe loss syndrome. The disease is caused by the proliferation of Human Immune Deficiency Virus (HIV) in the hosts’ body. HIV destructs the immune system of the body [**1**]. AIDS is a description of a disorder in the cellular and humoral immunity due to the infection with HIV. The main target of this virus is the T lymphocyte, which contains CD4 receptors in their cellular membrane. The disease varies from a mild viremia to a severe immune deficiency along with life-threatening opportunistic infections and even AIDS-related malignancies [**2**].

The original case of AIDS was described in 1980 in the United States between a group of homosexual men infected with Kaposi’s sarcoma and pneumocystis pneumonitis [**3**,**4**]. 15 years after the first AIDS case, pandemic HIV infection increased worldwide and in the late 1995, there were 1.3 million HIV-infected individuals among 193 countries. It is estimated that 24 million adults and 1.5 million children are infected with HIV and about 10000 new cases are added annually. As a result, the rising trend of HIV infection was continued in Sub-Saharan Africa and South Asia. The incidence rate of AIDS disease is of 2.5 million per year worldwide [**5**] and the incidence rate of HIV infection is of 19 cases per year [**6**]. 

HIV was transferred by heterosexual and homosexual relationship, blood transfusion and products, drug injection, infected pregnant mother, and finally infection of the neonate during childbirth, prenatal period, or breastfeeding. Needle stick injuries or penetration of sharp objects are transmission methods via skin and mucosal ways, and also sprinkle of blood and other body discharges into the eye, nose, and mouth [**7**-**9**]. AIDS is the cause of 25 million deaths worldwide and it was calculated that about 40 million affected patients do not have access to anti-retroviral treatment [**10**].

Assessments demonstrate that several factors can increase the risk of epidemic HIV which is the following: first, the prevalence of sexual transmitted diseases (STD) is partially high, which demonstrate the unprotected extramarital sex [**11**]. Also, fight, shift, and movement that are mostly accompanied by high-risk sexual behaviors that can increase the susceptibility of AIDS. Thirdly, injection drug users (IDUs) are a way for HIV transmission among the public population in some lands [**12**], and out of other factors, sexual contact with multiple partners, not using condom consistently, lack of information regarding HIV risks, and negative attitudes toward safe sexual function can also be pointed [**13**].

During the new ages, this disease was at this head of the health emergencies in Iran. Based on World safety community (WHO) forecasts, the incidence of HIV disease in Iran will be of 10 percent in 2020. Therefore, Iran is recognized as one of the riskiest countries in universe [**14**]. Epidemic HIV is spreading rapidly with different forms among various countries. In the current conditions, the composition of defensive and healing methods that emphasize on convenient access to these methods, are being discussed. So far, limited studies have been conducted on the currency from AIDS among the public population in Iran and it seems that the importance of this issue is not considered adequately. According to the high importance of this illness and with reference to what has been said, we aimed to assess the currency of HIV among the residents Island Qeshm. 

## Method

Utilizing the chance, cluster sampling method, this cross-sectional research is carried out on 511 men and 989 women in Qeshm Island of the direction of Iran through 2013-2014. At the time of the implementation of the research, the total number of people existing in This Island based on the latest public capitation study was 130000. Our example size (n=1500) with applying the next method:

$$n=\frac{z_{1-\frac{\alpha }{2}}^{2}p(1-p)}{d^{2}}$$

The House Registry at common wellness markets is con¬sidered as a sampling framework. Any common health center covered a part section of the Island. 2 raised interviewers then attended the problems’ houses and presented them by data about the research and its purposes. Data are accumulated with applying a checklist so is produced based on related subjects and experts’ view. The checklist included demographic data (age, gender, marital status, residency, Literacy, travelling to a foreign country and history of running away from home) and risk factors (addiction, sexual contact, imprisonment, STD and Tattooing) for AIDS.

The addition measures consisted of doing a Qeshm citizen and giving permission. Cases that did not provide their approval or those that are not ready next 2 connection efforts have been done are eliminated from the subject and followed by the next ran¬dom problems. Applying this method, a whole of 50 batches of 1500 people were involved in the study.

Members are surveyed into their houses, and a survey on private data is performed with a skilled interviewer, for any case. Members are then invited to apply to the Wellness Promoting Study Center, and they are presented by an opening word for gore sampling. One day later the record, a ten mL specimen of venous Gore is collected in ethylenediamine tetra-acetic acid (EDTA) glasses, after tourniquet employment at the Wellness Promoting Study Center and then transported to the local lab. 

Gore examples experienced qualitative evaluations to evaluate the renewed gore sampling. After the separation of serum from blood samples in the local laboratory, by centrifugation, serums were frozen in -20°C and transported to the central lab of the Iranian Gore Exchange Society. Serum examples are selected for HIV-Ab with ELISA though applying a third generation Kit (Biomatrix, Amsterdam). Positive samples are extracted for western spot test to be analysis. 

The research is accepted by the Humane Commission of the Medical Sciences Hormozgan University. A signed data approval is received from all of the members, and special data are stored private both as and after the research.

 The Received information is recorded in SPSS v.16 software and explained with applying detailed statistics (frequency, determine, percent, and regular variation) and chi-square analysis.

## Results

In the running research and to evaluate the seroprevalence of HIV-Ab, serum samples of 1500 subjects were analyzed for positivity with applying the ELISA method. None of samples was positive for HIV and all subjects are well. The base years of the members in this research was 31.35 years. From the studied subjects, 34.1 percent (n=511) and 65.9 (n=989) percent were male and female, respectively. 24.8 (n=372) and 75.2 (n=1128) percent of the members exist in towns and villages, respectively. 66% (n=990) were under diploma, 21.7% (n=325) had a diploma, 1.9% (n=29) had an associate degree, 8.2% (n=123) had a bachelor degree, and 2.2% (n=33) had a master degree and higher. 136 (7.9) participants had previously traveled to a foreign country and 1364 participants (92.1%) did not travel to a foreign country. None of the participants reported a history of running away from home (**Table 1**).

**Table 1:** Demographic data of participants

Variable	No.	Percent
Sexuality		
Men	510	38.1
women	988	65.9
Marital status		
Single	180	12
Married	1320	88
Residence		
Urban	372	24.8
Rural	1128	75.2
Literacy		
under diploma	990	66
diploma	325	21.7
associate degree	29	1.9
bachelor	123	8.2
master degree and higher	33	2.2
travelling to a foreign country		
Yes	137	7.9
No	1363	92.1
history of running away from home		
Yes	0	0
No	1500	100

According to this detection into **Table 2**, three participants had an addiction history and 1497 participants (99.8%) reported no addiction history. 0.3 percent (n=4) had a previous history of sexual contact and 2 participants had a prison history. 2 percent (n=34) had a history of STD and 98 percent (n=1466) reported no STD history. 4 percent (n=69) had a tattoo history and 96 percent (n=1431) of the participants reported no previous tattoo history. 

**Table 2:** Risk agents connected by Aids disease

parameter	No.	Percent
addiction history		
Opium	2	0.1
Heroin	2	0.1
Others	0	0
None	1497	99.8
Records of sexual connection		
Yes	5	0.3
No	1497	99.7
History of imprisonment		
Yes	2	0.1
No	1498	99.9
History of STD		
Yes	34	2
No	1466	98
History of Tattooing		
Y	69	4
N	1431	96

## Discussion

The HIV prevalence was zero in the now research. In a research managed with Estaminet al. [**15**], in 2009, among the health center clients in Andimeshk, the HIV prevalence was also zero. HIV prevalence was also zero in the study of Haghsehnaset al. [**16**] on prisoners. Similarly, the HIV prevalence was zero in the study of Nabavizadehet al. [**17**] in 1999 among blood donors of Yasuj. A retrospective cross-sectional was also conducted by Salehiet al. [**18**] on medical documents of blood donor volunteers during 2002 and 2005 in Isfahan and the events showed that the HIV currency is zero alike to the one in our research. In the research of Sharifiet al. [**19**] on the dentists of Qazvin city, the HIV prevalence was zero. No HIV positive case was reported in the study of Ghafoorian-Broujerdniaet al. [**20**] on medical documents of thalassemia patients referring to Shafa Hospital of Ahvaz during 1999 and 2004. In another study conducted by Kasraeianet al. [**21**] on blood donor volunteers of Shiraz Blood Transfusion Organization during 1998 and 2002, the HIV prevalence was 5.5 percent which was not compatible by the outcomes of our research. In the education of Kolivand [**22**] in Kermanshah, Taheri [**23**] in Rasht, and Masaeli [**24**] in Isfahan, the currency of Aids is 0.05, 0.008, and 0.018 percent, respectively, which did not match the results of our study. Similar studies were performed in Italy [**25**] and Usa [**26**], which demonstrated a significant decrease from 1995 to 2002, which can be ascribed the change of life style. There was no positive HIV case among the public population in the existing research, which can be expected educational and religious matters checking high-risk operations.

In the study of Bagheriet al. [**27**] on 1461 patients with AIDS, 819 (56.5 percent) patients had a past records of tattoo. This fact implies that tattooing is one of the substantial popular methods for HIV transmission. However, among 69 (4.4 percent) participants of our study by an earlier records of tattooing, the HIV prevalence was zero. In this research of Ghanbarzadehet al. [**28**] on the HIV prevalence among 199 female prisoners of Birjand, the HIV prevalence among 76 (38.2 percent) prisoners by a past history of tattooing was zero, that is consonant by the results of the current study. In the study of Dolan et al., although the tattooing prevalence was 30.1 percent, HIV prevalence was reported to be zero, which was congruent with the results in the present study. Different tattooing prevalence could be ascribed cultural and religious issues in this research area. 

The frequency of prison history among the HIV positive patients in this Keramatet research al. [**29**] was 40.4 percent. In the study of Strazzaet al. [**30**], the HIV prevalence among female prisoners was reported to be 13.9 percent. Consequently, residing in prison is one of the principal risk factors due to inappropriate health conditions, malnutrition, higher affinity to drug abuse through injection, high-risk sexual behaviors, and as a result a higher probability of HIV transmission. In the running research, 2 members had previous history of being in prison; but, all are not HIV positive. Those members by a past records of doing in prison, which was one of that main risk factors for HIV was limited in our study, which implied an ethical health of our community and was consistent with the studies of Nokhodian [**31**] and Mohamed [**32**] who reported the HIV prevalence to be equal to zero among prisoners. 

In the study of Robinson et al. [**33**], 90 percent of the HIV positive cases were also affected by other STDs. Lagaet al. [**34**] demonstrated that the STD is a major danger factor for HIV and the annual HIV incidence was 9.8 percent before the STD control, which approached to 4.8 percent following the STD control. Ghyset al. [**35**] revealed that the HIV incidence rate decreased from 16.3 percent to 6.5 percent following the STD control. The results of these studies verified that the STD is another a major danger factor for HIV transmission. The HIV currency is zero in the available research, although 34 (2 percent) participants reported a previous history of STD. In the study of Ghanbarzadehet al. [**28**] on female prisoners of Birjand, the HIV prevalence was reported to be zero despite the high prevalence of STD, which could be due to the early visits to physicians, rapid Determination, and proper and appropriate way. As stated previously, the HIV prevalence can be reduced through STD control. 

Moradiet al. [**36**] conducted a study in 1998 which assessed the seroepidemiology of AIDS in Iran. Medical documents of 1953 patients with AIDS were assessed and results demonstrated that 30.1 percent of them had previously traveled to a foreign country. HIV prevalence was zero in the current study, although 136 (7.9 percent) participants had previously traveled to a foreign country. This can be like greatest of the members in the existing research are coupled, that can be a cause for restricting high-risk sexual habits.

## Conclusion

The most of members in the existing research lived in rural areas and were married women with below diploma education level. The currency of Aids was zero, despite the previous history of STD and tattooing. Collectively, more efforts were required to raise the public awareness regarding the risks of tattooing and the education regarding the prevention of AIDS and another sexually spread illnesses. 

**Limitations**

 The following are the restriction of the research:

1- Lack of cooperation the studied society in presenting the required information

2- Unavailability of scientific resources

3- Cost 

**Acknowledgements**

This research is the outcome of a general physician thesis. We would want to thank all professors, Qeshm residents, health system officials, and Medical Sciences Hormozgan University that supported us through the research.
